# Cardiac Autonomic Modulation Response Before, During, and After Submaximal Exercise in Older Adults With Intellectual Disability

**DOI:** 10.3389/fphys.2021.702418

**Published:** 2021-10-15

**Authors:** Manel Font-Farré, Ana Claudia Silva Farche, Anielle C. de Medeiros Takahashi, Myriam Guerra-Balic, Arturo Figueroa, Guillermo R. Oviedo

**Affiliations:** ^1^Faculty of Psychology, Education and Sport Science Blanquerna, University Ramon Llull, Barcelona, Spain; ^2^Department of Physiotherapy, Federal University of São Carlos, São Carlos, Brazil; ^3^Department of Kinesiology and Sport Management, Texas Tech University, Lubbock, TX, United States; ^4^School of Health Science Blanquerna, University Ramon Llull, Barcelona, Spain

**Keywords:** intellectual disability, cardiac autonomic modulation, heart rate variability, older adults, physical activity, heart rate kinetics

## Abstract

The analysis of the heart rate variability (HRV) consists of changes in the time intervals between consecutive R waves. It provides information on the autonomic nervous system regulation and it is a predictor of adverse cardiovascular events. Several studies analyzed this parameter in youth and adults with Intellectual Disability (ID). Nevertheless, there is a lack of information regarding the HRV before, during, and after exercise in older adults with ID. Therefore, we aimed to describe and compare the cardiac autonomic modulation before, during, and after the six-minute walk test (6MWT) in older adults with and without ID. Twenty-four volunteers with ID and 24 without ID (non-ID) participated in this study. HRV was assessed by R-R intervals at rest, during and after the 6MWT. At rest and recovery periods, the participants remained sited. The symbolic analysis was used to evaluate non-linear HRV components. The recovery HR kinetics was assessed by the mean response time, which is equivalent to time constant (τ)+time delay (TD). Between groups differences in HRV variables were not significant. During the recovery period, HR kinetics time variables showed significant better results in non-ID participants (TD: 6±5s vs. 15±11s; τ: 19±10s vs. 35±17s; and MRT: 25±9s vs. 50±11s, all *p*<0.050). In conclusion, our results suggest that the HRV in older adults with and without ID is similar during rest, exercise, and recovery. Recovery HR kinetics after the 6MWT was slower in older adults with ID. The reason for these results may be a reduced post-exercise vagal rebound in older adults with ID.

## Introduction

Persons with intellectual disability (ID) are characterized by having significant limitations in both intellectual functioning and maladaptive behavior, and it originates before the age 22 (Schalock et al., 2021). This disability may affect the nervous and/or the sensory systems, can cause metabolic and degenerative disorders, and may result in deficits in functioning and physical disability (Harris and Greenspan, 2016). The total number of older people with ID is increasing, as well as their life expectancy, because of improved healthcare (Patja et al., 2000). However, several reasons indicate a risk of low physical fitness levels in this population and a higher risk of suffering hypertension, hyperinsulinemia, dyslipidemia, and obesity (Rimmer and Yamaki, 2006) and developing chronic multimorbidity when compared to the general population (Rimmer and Yamaki, 2006; Hermans and Evenhuis, 2014; Cooper et al., 2015, 2020).

The metabolic syndrome is a group of factors that increases the risk of suffering cardiovascular diseases and type 2 diabetes. These factors are as: insulin resistance, abdominal fat, and atherogenic dyslipidemia, among others (Alberti et al., 2009). The different components of the metabolic syndrome have been related with lower heart rate variability (HRV; Stuckey et al., 2015).

The analysis of the HRV consists of changes in the time intervals between consecutive R waves. It is a noninvasive measure of cardiac autonomic modulation which is usually measured as the standard deviation of the mean R-R intervals (RRi) of all cardiac cycle length and can be assessed by many different approaches (Shaffer and Ginsberg, 2017; Silva et al., 2017). Healthy people present a high degree of HRV which allows the cardiovascular system to respond quickly and efficiently to changes in blood pressure (Shaffer and Ginsberg, 2017). Decreased HRV is related to a number of risk factors (e.g., obesity, dyslipidemia, and hypertension) for CVD (Britton et al., 2007; Thayer et al., 2010; Hillebrand et al., 2013). In people with ID, HRV is also influenced by factors like obesity, low physical fitness, and age (Mendonca et al., 2013).

Different studies have shown that there is an autonomic dysfunction in people with ID when analyzing the HRV at rest and during exercise (Baynard et al., 2004; Chang et al., 2012) and deficits in the chronotropic response to isometric handgrip exercise in adults with ID (Dipla et al., 2013). Hilgenkamp et al. (2021) studied the autonomic response to standing up (active orthostasis) and head-up tilt position (passive orthostasis) in individuals with ID compared to a control group without ID. The authors found that individuals with ID presented altered hemodynamic and autonomic regulation to the clinical autonomic function tasks standing up and head-up tilt, a higher resting heart rate and higher mean arterial pressure, which suggested a higher arousal level and a blunted response in parasympathetic modulation (Hilgenkamp et al., 2021). Another study assessed autonomic nervous system function in young adults with ID (aged 18–45yr), analyzing the sudomotor function, heart rate, and systolic blood pressure variability, as well as their cardiac baroreflex function. Their results showed that there is an increase of cardiovascular risk markers and autonomic dysfunction in this population with intellectual disability compared with a control group without ID (Zwack et al., 2021). Moreover, as worse level of ID, the autonomic dysfunction was more marked (Zwack et al., 2021).

If we focused specifically in Down Syndrome (DS) individuals, there is an autonomic dysfunction in individuals with DS, which may or may not be expressed at rest (Dias de Carvalho et al., 2018). Some authors reported attenuated HR and HRV during and after isometric handgrip exercise in young adults with DS compared with peers without disabilities (Figueroa et al., 2005). On contrary, various studies did not found differences in the HRV between ID and non-ID persons at rest (Baynard et al., 2004; Mendonca et al., 2011; Chang et al., 2012).

Nevertheless, there are no studies analyzing the cardiac autonomic response during and after a stressful stimulus, such as physical exercise in seniors with ID. When exercise stops, both HRV and HR usually demonstrate a time-dependent recovery and eventual return to pre-exercise levels, depending on exercise duration and intensity (Michael et al., 2017). Therefore, in the post-exercise recovery period, the cardiac autonomic response is not only studied by HRV but also through the analysis of the decay-time constant of the off transient phase of HR (t-off) kinetics.

The HR t-off kinetics is a practically exponential downfall (Storniolo et al., 2020) modulated by the interaction of multiple factors related to parasympathetic reactivation and sympathetic withdrawal (Pierpont and Voth, 2004). Thus, HR t-off kinetics can assess the parasympathetic reactivation following exercise (Junior et al., 2019). Moreover, it is known that HR recovery following exercise occurs more rapidly in individuals with greater aerobic fitness (Michael et al., 2017).

HR t-off kinetics has been shown to provide clinical information (Javorka et al., 2003) on autonomic impairment during post-exercise periods and be predictors of mortality and increased risk of cardiovascular events (Morshedi-Meibodi et al., 2002). Abnormalities in t-off kinetics have been reported in type 2 diabetes (Baldi et al., 2016), obesity (Franco et al., 2015), and aging (Simões et al., 2013). In people with ID, there is an attenuation in the HR recovery, especially in DS (Figueroa et al., 2005).

Autonomic function is also affected by aging. Some authors conclude that there is a decline in HRV (Soares-Miranda et al., 2014; Almeida-Santos et al., 2016; Rastović et al., 2019) and heart rate recovery (HRR; Simões et al., 2013) with age. This occurs because of the reduction in the efferent cardiovagal tone and the changes in beta-adrenergic response, which shift the autonomic balance toward sympathetic dominance and cardiovascular dysfunction (Leosco et al., 2013). People with ID age prematurely showing early signs of aging in their 40s and 50s. Consequently, the prevalence of poor physical fitness, musculoskeletal disability, visual impairments, and metabolic syndrome factors is similar to people older than 60years of age without ID (Novell et al., 2008; Berjano-Peirats and García-Burgos, 2010; Lin et al., 2011).

As far as we know, no previous studies have examined cardiac autonomic modulation during and after an activity of daily living in older adults with ID. Therefore, the present study aimed to assess and compare the cardiac autonomic modulation using the HRV response during and after a submaximal aerobic exercise test (6MWT) and the t-off HR kinetics after the test in older adults with and without ID.

## Materials and Methods

### Study Design and Participants

This is a cross-sectional study that used data from participants that were recruited from a convenience sample from two occupational day centers for people with ID and one from community-dwelling older adults without ID (non-ID). A total of 24 older adults with mild to moderate ID without DS and 24 non-ID older adults volunteered to participate in the study. Inclusion criteria were as: a) non-ID participants aged ≥60years, and ID participants aged ≥45years; b) a normal 12-lead electrocardiogram (ECG) at rest; c) being able to perform the 6-min walk test (6MWT) without external aids; and d) willing to provide a written consent or from the tutor/legal guardian for the non-ID and ID participants, respectively, as well as an informed assent adapted for the last ones.

Exclusion criteria for both groups were to have as: (a) cardiac arrhythmia; (b) a pacemaker; (c) unstable angina; (d) suffered a myocardial infarction; (e) contraindications to exercise; (f) vestibular and visual disorders that may influence the assessments; (g) use of medications that may influence HR and/or response to exercise; (h) been diagnosed with severe or profound ID; (i) been diagnose with DS; (j) inability to communicate orally; (k) inability to provide written informed consent; and (l) parents/legal tutors not willing to provide written informed consent.

This study was approved by the Institutional Review Board (CER URL 2017_2018_008 and CEP UFSCar 2016/1.800.231) and complies with the principles of the Declaration of Helsinki (World Medical Association, 2013).

#### Participants With ID

Occupational centers for adults with ID from Barcelona were contacted to be part of the present study. The research team informed professionals about the background and the aims of the study. The contact person of each center further received all information about the recruitment strategy (inclusion criteria and exclusion criteria).

In the first meeting, the researchers explained the study protocols to interested participants with ID and their families/legal tutors and gave them an information sheet about the study. Prior to participation, all participants and parents/legal tutors signed an informed consent and assent.

#### Participants Without ID

Community-dwelling older adults without ID were recruited by social media and health centers. The principal researchers explained the trial to the interested participants, gave them an information sheet about the study, and obtained the participants’ informed consent to be included in this study.

### Testing Procedure

Before the testing, all the participants had familiarization sessions to become acquainted with the experimental protocols, equipment, and techniques used in the study. Participants were requested to not engage in moderate or vigorous exercise the day before testing, to be fasted for at least 4h, and did not consume alcohol and/or caffeine for at least 12h before the assessments.

After signing the informed consent and assent, all participants were submitted to a structured anamneses and questionnaire that included information about age, sex, co-morbidities, and medicine use.

#### Anthropometric Measurements

Height was measured to the nearest 0.1cm using a stadiometer (Seca 225, Seca, Hamburg, Germany). Weight was measured to the nearest 0.1kg on a digital scale (Tanita MC-780U, Arligton Heights, IL, United States) with the participant wearing lightweight clothing and no shoes. Body mass index (BMI) was calculated as weight in kilograms divided by height in squared meters (kg/m^2^).

#### Six-Minute Walk Test

The 6MWT is used to assess the submaximal exercise capacity of individuals from the longest distance walked in 6min (ATS Committee on Proficiency Standards for Clinical Pulmonary Function Laboratories, 2002). For the 6MWT, the recommendations were followed according to the American Thoracic Society (2002). During the test, participants have to walk at a self-paced velocity in a 30m unobstructed corridor. The participants walked as fast as they could (without running) and as far as possible. Standardized phrases for encouragement were used during the test (ATS Committee on Proficiency Standards for Clinical Pulmonary Function Laboratories, 2002). The reliability and validity of this test for persons with ID were assessed in a previous study (Guerra-Balic et al., 2015).

#### Heart Rate Signal Register

Heart rate and interbeat intervals were recorded beat-to-beat using a HR monitor (Polar^®^ RS800CX, Polar Electro OY, Finland) at a sampling frequency of 1,000Hz and all data were downloaded using the ProTrainer System Software (Polar^®^ ProTrainer 5, Polar Electro OY, Finland). The RRi were registered at rest in a sitting position for 10min; during the 6MWT; and through the 10-min recovery period on sitting position.

#### Heart Rate Variability Analysis

The cardiac autonomic modulation was evaluated by using the non-linear parameters of the HRV. Two hundred and fifty-six RRi sequences with the greatest stability were chosen for sitting rest, 6MWT, and sitting recovery phases (Task Force of the European Society of Cardiology and the North American Society of Pacing and Electrophysiology, 1996). Evident non-stationary series, as well as progressive increases or decreases or sudden variance changes, were excluded (Magagnin et al., 2011).

Spectral analysis is a linear method based on the calculation of power spectral density which usually analyses the low-frequency (LF) to high-frequency (HF) power oscillation ratio (LF/HF; Pagani et al., 1986). Nevertheless, different authors explain that there are methodological drawbacks when using spectral analysis, as very segmented results by the frequency bands definition (Malliani et al., 1998; Porta et al., 2007a). The non-linear analysis of the HRV, named symbolic analysis, overcomes the limitations of the linear analysis (Porta et al., 2001, 2007a; Tobaldini et al., 2009; Perseguini et al., 2011; Silva et al., 2017). Therefore, the sympathetic and parasympathetic components were presented using symbolic analysis, which provide more stable results and can minimize abnormalities and alterations that are otherwise not apparent, generated by frequency band definition (Porta et al., 2007b; Takahashi et al., 2012). In this context, studies suggest that non-linear methods are able and more appropriate for characterize the dynamics and degree of recurrence of a temporal pattern (Lipsitz, 2002; Huikuri et al., 2009; Takahashi et al., 2012).

The symbolic analysis was based on the Uniform Quantization Process described by Porta et al. (1998). It was carried out by six quantization levels and grouping the patterns with three symbols into four families as follows: (a) no variation (0V); (b) one variation (1V); (c) two like variations (2LV); and (d) two unlike variations (2UV). The rate of occurrence for each pattern was defined as 0V%, 1V%, 2LV%, and 2UV% (Porta et al., 2001, 2007a).

#### Heart Rate Off-Kinetics Analysis

The t-off HR kinetics of the recovery period was analyzed by using the CardioKin 1.2 software routine, according to the parameters calculated by a certified LabVIEW-associated developer (LabVIEW 2012, National Instruments, Austin, TX, United States). The model used for fitting the kinetic response in the exercise-recovery transition was based on previous studies (Rossiter et al., 2002; Beltrame et al., 2018). The HRR dynamics (dependent variable) was modelled using the following exponential function (Rossiter et al., 2002): HRR(t)=HRpeak – *a* ^*^ (1−e^-(t-TD)/τ^).

The independent variable “t” is time (recovery time), “HRpeak” is the peak five-sample average HR at the end of the 6MWT, and “*a*” is the magnitude of change between the HRpeak and the steady-state HR at the end of the recovery period. The time constant “τ” (i.e., the speed of HRR dynamics) is defined as the time for the HR to decrease to 63% of the final amplitude “*a*” after a given time delay “TD” considered as the parameter that represents a delay in the response time on the recovery period (Rossiter et al., 2002; Beltrame et al., 2018). The overall kinetics of HR was determined by the mean response time (MRT), which was used to indicate the rate of change of the HR toward the new steady-state (Whipp et al., 2005). Thus, the MRT for a single term exponential model is equivalent to τ+TD (Hughson and Morrissey, 1983). We analyze the t-off HR kinetics during 10min after the end of the 6MWT on sitting position.

### Statistical Analysis

Descriptive statistics were obtained for all variables. The Shapiro-Wilk test was used to verify the normality of data distribution. Chi-square test was used to investigate differences in proportions between sex and groups, while independent-samples *t*-tests were used to compare means.

A 2×3 [group (2: ID and non-ID) × time (3, rest, 6MWT, and recovery)] repeated measures ANOVA was performed to compare HRV parameters (mean RRi, variance, 0V%, 1V%, 2LV%, and 2uv%) between- and within-groups. Significant between-group differences at each level were examined using independent-samples *t*-tests, whereas within-group effects were examined using paired-samples *t*-tests. All models included age and BMI as covariates, and Bonferroni adjustments were used for comparisons. Between groups t-off HR kinetics variables were analyzed using independent-samples *t*-tests.

The critical values for statistical significance were assumed at an alpha level≤0.05. Statistical analyses were conducted using Statistical Package for the social sciences (IBM SPSS, v 22.0, Chicago, IL, United States).

## Results

### Participants’ Characteristics, Anthropometrics, and Walk Distance

A total of 48 participants (60.38±7.5years old) provided data for the study. Participants’ characteristics and submaximal aerobic capacity are summarized in Table 1.

**Table 1:** Participants’ characteristics, anthropometry, and submaximal aerobic capacity.

Variables	Non-ID group (*n*=24)	ID group (*n*=24)	*p*
	Mean (SD)	Mean (SD)	
**Characteristic**			
Age (y)	66.0(4.2)	54.7(5.6)	<0.001
Sex (m/f)	9/15	15/9	0083
**Anthropometrics**			
Weight (kg)	67.6(11.2)	76.2(13.0)	**0.017**
Height (m)	1.61(0.08)	1.62(0.08)	0.755
BMI (kg/m^2^)	25.9(3.9)	29.1(5.2)	**0.021**
**Submaximal aerobic test**			
6MWT (m)	517.0(115.0)	493.0(60.4)	0.742
HR rest (bpm)	74.4(9.9)	74.8(14.6)	0.785
HR peak (bpm)	109.1(14.8)	124.6(20.1)	**0.026**
Percentage of predicted maximal HR (%)	71.0(10.3)	76.0(11.9)	0.123
HR recovery (bpm)	89.8(19.3)	85.2(14.1)	0.761

*Values are expressed as mean (standard deviation) or total of individuals (percentile). ID, intellectual disability; y, years; m, male; f, female; BMI, body mass index; HR, heart rate; and 6MWT, 6-min walk test. Statistically significant values are showed in bold (p≤0.05)*.

Non-ID participants were older than ID participants (66.0±4.2 vs. 54.7±5.6years; *p*<0.001). ID participants were heavier and had a larger BMI than the non-ID volunteers (*p*= 0.017 and *p*=0.021, respectively).

During the 6MWT, both groups walked similar distances (517.0±115.0m vs. 493.0±60.4m; *p*=0.742). The ID participants reached significantly higher peak HR values during the 6MWT than non-ID participants (*p*=0.026).

### Autonomic Modulation

Table 2 presents the HRV analysis on rest, 6MWT, and recovery periods. Between groups differences in HRV variables were not statistically significant.

**Table 2:** Mean, variance, and symbolic analysis in older adults with and without ID.

Variables	Non-ID group (*n*=24)			ID group (*n*=24)			*p*		
	Mean (SD)			Mean (SD)			Group	Moment	Interaction
**Linear Parameter**	**Rest**	**6MWT**	**Recovery**	**Rest**	**6MWT**	**Recovery**
Mean RRi (ms)	895.3(148.0)	699.5(138.8)	858.3(162.6)	850.0(169.6)	689.8(190.8)	803.8(144.6)	0.247	0.970	0.232
Variance (ms)	311.9(400.2)	431.9(420.3)	564.5(483.7)	892.2(152.6)	548.6(435.0)	679.8(723.6)	0.051	0.450	0.066
**Symbolic analysis**									
0V%	24.5(13.2)	27.4(15.0)	32.5(17.8)	33.3(15.8)	32.8(12.7)	34.2(14.8)	0.524	0.710	0.667
1V%	33.7(6.1)	41.0(9.2	32.3(10.5)	31.9(5.5)	36.3(6.5)	32.4(6.0)	0.113	0.281	0.200
2LV%	22.4(4.0)	17.6(3.9	17.4(5.7)	21.3(3.7)	15.5(28)	17.5(3.2)	0.166	0.758	0.393
2UV%	19.4(11.2)	14.1(8.9)	17.9(12.0)	13.5(8.9)	15.4(11.3)	16.0(8.5)	0.593	0.754	0.318

*Values are means (standard deviation). ID, intellectual disability; 6MWT, 6-min walk test; and RRi, R-R intervals*.

### Heart Rate Off-Kinetics

Table 3 presents the HR kinetics on the recovery period. During this phase, time variables showed significant faster t-off HR kinetics in non-ID participants than in ID participants (TD: 6±5s vs. 15±11s, *p*=0.001; τ: 19±10s vs. 35±17s, *p*=0.001; and MRT: 25±9s vs. 50±11s, *p*<0.001).

**Table 3:** Heart rate kinetics during recovery from 6MWT in older adults with and without ID.

Variables	Non-ID group (*n*=24)	ID group (*n*=24)	*p*
	Mean (SD)	Mean (SD)
**Heart Rate Kinetics – t-off**			
*a* (bpm)	17(6)	20(7)	0.152
TD (s)	6(5)	15(11)	**0.001**
*τ* (s)	19(10)	35(17)	**0.001**
MRT (s)	25(9)	50(11)	**<0.001**

*Values are means (standard deviation). ID, intellectual disability; a, heart rate amplitude; TD, time delay; τ, time constant; and MRT, mean response time. Statistically significant values are showed in bold (p≤0.05)*.

**Figure 1:**
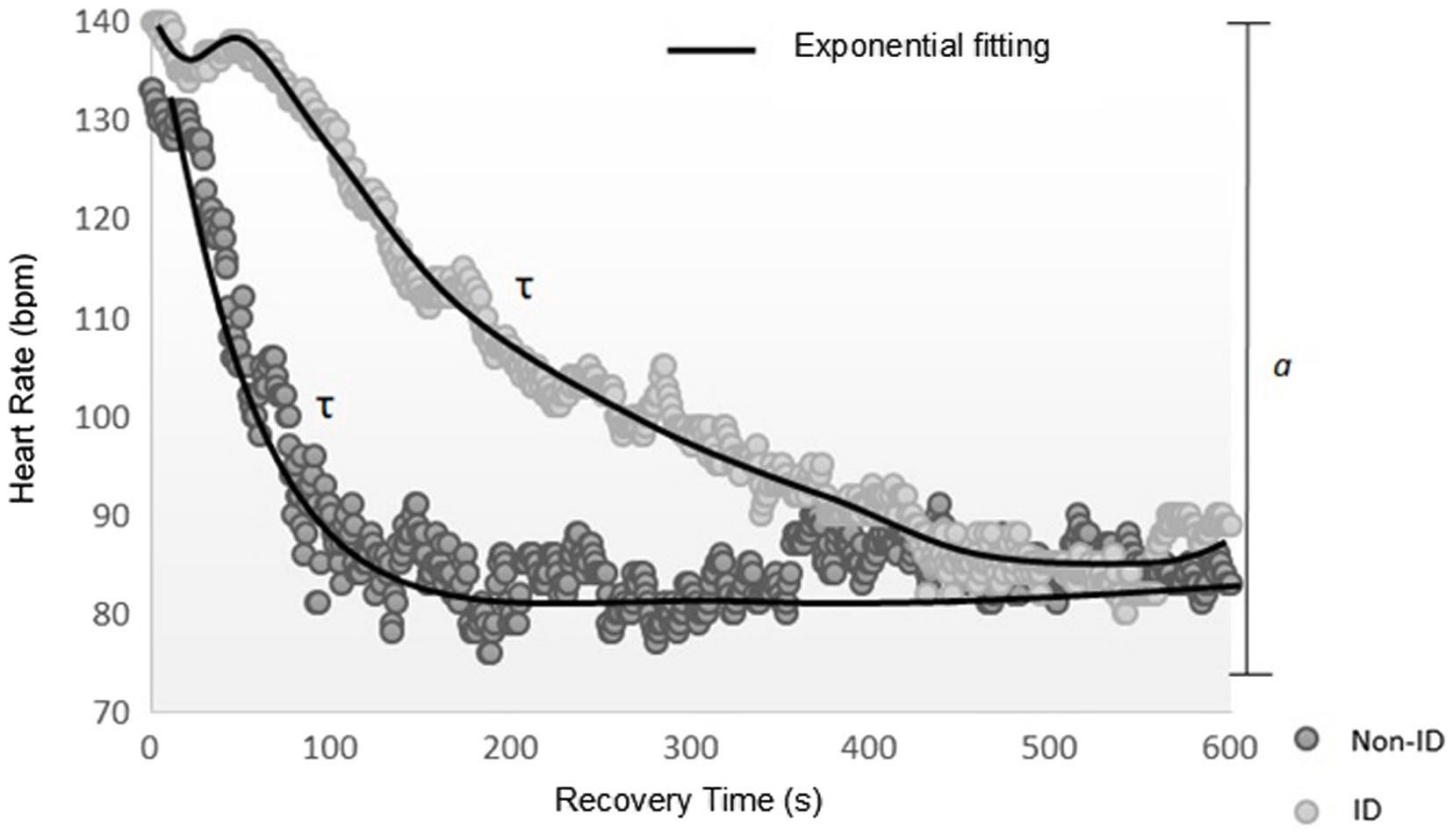
Representation of the HR recovery response after the 6-min walking test in one participant with ID and a non-ID participant. Data were fitted by an exponential function to characterize the dynamic response of HR recovery. HR, heart rate; τ, time constant; *a*, heart rate amplitude; ID, older adult with intellectual disability; and Non-ID, older adult without intellectual disability.

Figure 1 shows the graphical signal of HR kinetics on recovery period of one ID volunteer and a non-ID volunteer.

## Discussion

The main purpose of the study was to assess and compare the HRV before, during, and after the 6MWT and the t-off HR kinetics during the recovery period in older adults with and without ID. The main findings were as: a) during the 6MWT, older adults with ID presented higher HR values than older adults without ID; b) HRV before, during, and after 6MWT was similar in both groups; and c) the HR t-off kinetics of the older adults with ID was slower than the group without ID.

### HR and Autonomic Function at Rest Conditions

Vagal modulation is the dominating control factor of HR at rest (Baynard et al., 2004). Our data suggest that, largely, this cardiovagal modulation at rest is similar in persons with ID than in peers without ID. Despite the ID volunteers had higher sympathetic activity (0V%: 33.3) during resting period when compared with non-ID participants (0V%: 24.5), the difference between groups was not significant when age and BMI were used as covariate.

Our data show that our participants with ID have similar values of HRV than the group without ID. Our results are consistent with previous studies that reported similar HRs and HRV in people without ID and in ID persons with and without DS (Figueroa et al., 2005; Iellamo et al., 2005; Goulopoulou et al., 2006; Agiovlasitis et al., 2011; Dipla et al., 2013). Even though, these findings are in contrast with those from Chang et al. (2012), who demonstrated that ID adults, especially those with metabolic syndrome, present alterations in cardiac autonomic activity at rest. Nevertheless, these authors analyzed HRV data from younger participants (men: 33.27±9.32yrs.; women: 32.66±8.54yrs.) than those in the present study. These authors perform the HRV assessment in the supine position and not in seated position. In addition, the data from the non-ID group that these authors used to compare with their ID volunteers were young adults with metabolic syndrome from the Cardiovascular Risk in Young Finns Study (Koskinen et al., 2009). Finally, the HRV at rest found in our participants with ID was similar than that reported for healthy older adults (Takahashi et al., 2012).

### HR and Autonomic Function in Response to Exercise

When exercise starts, there is an increase of metabolic demands including cardiac output, which is due to attenuated parasympathetic activity and increased sympathetic activity (Rosenwinkel et al., 2001). In our study, both groups showed a significant reduction on mean RRi values during the submaximal exercise, with a normal increase of HR at the beginning of exercise, which may be due to vagal withdrawal, and a gradual tachycardia, attributed to a sympathetic modulation (Soares et al., 2017). Some studies demonstrated that this behavior occurs not only in older adults, but also in healthy young and middle-aged people (Trevizani et al., 2015; Catai et al., 2020). Despite there is a tendency for the non-ID group to achieve greater sympathetic modulation (0V%) and the parasympathetic withdrawal (2UV%) during exercise, the differences between groups were not significant. This interpretation is similar than that of Baynard et al. (2004) who concluded that the autonomic modulation of HR appears to be appropriate for both ID without and with DS for submaximal aerobic exercise intensities, with no indication of cardiac autonomic dysfunction. Our findings are in contrast with those from Dipla et al. (2013), who concluded that individuals with ID present deficits in the chronotropic response to isometric handgrip exercise and a blunted metaboreflex-induced pressor response compared with non-ID individuals. In their study, however, they compared adult males with mild to moderate ID (26.4±0.5yrs) with non-ID participants (25.1±0.5yrs). They also performed a handgrip exercise (with and without occlusion) instead of a submaximal aerobic exercise, and they used the *Poincaré plot* analysis for the HRV analysis and not the symbolic ones. These differences in the age profiles, the test methodology, and the HRV analysis, together with the use of other autonomic modulation examinations (metaboreflex and hemodynamics), could explain why they have found significant differences and we have not.

After an initial stage, on which HR increases due to the inhibition of vagal activity, as one goes on exercising, HR increases again. In our study, older adults with ID reached higher exercise HR peak values than the non-ID group (25.5bpm more). So, it seems that the ID participants need a higher cardiovascular effort than the older adults without ID to achieve similar walking distance in 6min. This difference could not be explained by a greater workload, imposed by higher body weight, on the cardiovascular system during exercise because of the use of BMI and sex as covariates. Thus, our findings may suggest that lower HR achieved during the 6MWT by the seniors without ID is the result of different central and peripheral factors that allow them to perform the same work at lower energy cost (Fernhall, 1993; Lamberts et al., 2004; Hilgenkamp et al., 2012; Macinnis and Gibala, 2017).

### HR and Autonomic Function During Recovery Periods

In contrast to previous studies (Baynard et al., 2004; Chang et al., 2012; Dipla et al., 2013), we also examined the HR during the recovery period. In the present study, neither group was able to reach resting HR values (ID group: 74.8 vs. 85.2bpm; non-ID group: 74.4 vs. 89.8bpm) within 10min of recovery. Another study found that only 38.6% of older adults with ID without DS did not experience complete HR recovery 5min after they performed a 10-m incremental shuttle walking test (Oppewal et al., 2014). In our study, HRV during recovery period was similar in both groups.

### HR Kinetics During Recovery Period

HR recovery after exercise is considered a strong predictor of mortality in adults without ID (Cole et al., 2000) and is related with vagal tone abnormalities and low physical fitness levels (Imai et al., 1994). The decline of HR after cessation of exercise is the variable most commonly analyzed to assess parasympathetic reactivation and sympathetic deactivation (Rosenwinkel et al., 2001; Peçanha et al., 2014). The initial HR decay is strongly dependent on parasympathetic reactivity (fast phase), meanwhile the sympathetic withdrawal occurs after the first minute of the recovery (slow phase; Peçanha et al., 2014). For this reason, the measure of HR recovery after exercise allows us to investigate the cardiovagal tone (Rosenwinkel et al., 2001).

In our study, despite both groups have a similar HR recovery amplitude (*a*), ID participants took longer than non-ID participants after the exercise to recover almost the same HR [MRT(s): 50s vs. 25s, respectively]. This occurred not only because the ID group spent more time between the end of the test and the start of the decrease in HR (TD: 15s in ID group vs. 6s in non-ID group), but also because their slower HR decrease to the 63% of the final amplitude “*a*” after a given time delay “TD” (τ: 35s in ID group vs. 19s in non-ID group). The parasympathetic withdrawal with the onset of exercise is also well established in the literature (Baynard et al., 2004). Figure 1 shows the differences in the t-off HR kinetics between one participant with ID and one non-ID peer, where it can be seen that the non-ID participant has a faster and more efficient t-off HR kinetics than the ID participant.

During the recovery phase, most of the difference between groups in the reduction of HR occurs at the beginning of the recovery period. This goes in line with Oppewal et al., 2014, which concludes that the largest decrease in their sample’s HR was in the first minute of recovery. The authors analyzed the HRR after a 10-m incremental shuttle walking test in persons with ID aged older than 50years with and without DS. The HR peak of the non-DS participants (≈119bpm) was similar than the HR peak of the ID participants in our study (≈125bpm) and the time performing the test, despite it was not the same, was also similar (Oppewal et al. (2014): 316s vs. our study: 360s). The principal differences between both studies are the age of the samples (60.5±7.3yrs. vs. 54.7±5.6yrs), the recovery period (5min vs. 10min), the exercise intensity (maximal vs. submaximal), and the HR analysis (HRR vs. t-off HR kinetics). Despite these differences, on both studies, the main changes in HR after exercise occurred at the beginning of the recovery phase. Unfortunately, the methodological differences between the two studies do not allow us to draw further conclusions from this comparison.

The faster t-off HR kinetics in non-ID older adults together with the non-significant differences in HRV could be explained by a release of inhibitory commands from the motor cortex to the parasympathetic center, producing an impairment in the mediation of the vagal rebound (Imai et al., 1994), which is independent of the sympathetic withdrawal (Figueroa et al., 2005). These results are in consonance with Figueroa et al. (2005), who reported attenuated HR, vagal rebound, and systolic blood pressure after handgrip exercise in young adults with DS.

When symbolic analysis based on the Uniform Quantization Process described by Porta et al. (1998) is used for the analysis of HRV in the recovery phase, it always needs at least 2min to find the 256 RR intervals (RRi) sequences with the greatest stability (Task Force of the European Society of Cardiology and the North American Society of Pacing and Electrophysiology, 1996). Therefore, we believe that it would be more appropriate to use the t-off HR kinetics to analyze the autonomic modulation during the recovery phase as it seems to be more sensitive to detect the initial changes of HR after submaximal exercise.

### Limitations

It is important to acknowledge the limitations of this study. Firstly, we have selected a control group of adults without ID ≥60years old because adults with ID aged ≥40–45 present health problems and co-morbidities similar to that of their non-ID peers aged 60 or older (Novell et al., 2008; Berjano-Peirats and García-Burgos, 2010). For these reasons, age was used as covariate on all statistical models.

Secondly, as reported by different studies (Sinnreich et al., 1998; Zhang, 2007), there is an important effect of sex on HRV. Therefore, the unequal number of men and women in our study should be taken into account as a possible limitation. Nevertheless, in this study, between groups differences in the proportion of women and men were not significant.

Finally, interpretation of these results should be treated with caution, owing the small sample of adults with and without ID included in this study. Therefore, more studies including different ID etiologies and levels will be necessary to gain a greater understanding of the HRV and t-off HR kinetics in this population, as well as, studies with ID and non-ID age-matched samples, despite our belief that the ID participants would probably have a lower functional capacity.

## Conclusion

We investigated cardiac autonomic function responses in seniors with and without ID and its responses during and after submaximal aerobic exercise. We found that the HRV in both groups was similar at rest and during exercise and recovery.

The t-off HR kinetics analysis showed that the HR recovery after a submaximal exercise is slower in persons with ID. These results may be due to a reduced post-exercise vagal rebound in older adults with ID.

## Data Availability Statement

The raw data supporting the conclusions of this article will be made available by the authors, without undue reservation.

## Ethics Statement

The studies involving human participants were reviewed and approved by the Comité de Ética e Investigación de la Universitat Ramon Llull. The patients/participants provided their written informed consent to participate in this study.

## Author Contributions

MF-F, AF, and GO contributed to the conceptualization, methodology, investigation, data curation, formal analysis, and writing original draft. AT, MG-B, and AF wrote, reviewed, and edited the original draft and contributed to the formal analysis. All authors contributed to manuscript revision, read, and approved the submitted version.

## Funding

This study was supported by the Spanish Ministry of Economy, Industry, and Competitiveness (I+D+i grant number: DEP2017–86862-C2–1-R) and by the Secretaria d’Universitats i Recerca del Departament d’Empresa i Coneixement de la Generalitat de Catalunya i la Universitat Ramon Llull (Ref: 2021-URL-Proj-042) and FI grant (ref: 2021 FI_B200162).

## Conflict of Interest

The authors declare that the research was conducted in the absence of any commercial or financial relationships that could be construed as a potential conflict of interest.

## Publisher’s Note

All claims expressed in this article are solely those of the authors and do not necessarily represent those of their affiliated organizations, or those of the publisher, the editors and the reviewers. Any product that may be evaluated in this article, or claim that may be made by its manufacturer, is not guaranteed or endorsed by the publisher.
